# Comment on “Expression of CD44 and the survival in glioma: a meta-analysis”

**DOI:** 10.1042/BSR20202812

**Published:** 2020-10-28

**Authors:** Chongxian Hou, Han Lin, Peng Wang, Yong Yang, Siyi Cen, Dong Zhou

**Affiliations:** 1 Department of Neurosurgery, Guangdong Provincial People’s Hospital, Guangdong Academy of Medical Sciences, Guangzhou 510080, China; 2 Medical College, Shantou University, Shantou 515041, Guangdong, China; 3 College of Pharmacy, South-Central University for Nationalities, Wuhan, China

**Keywords:** biomarker, CD44, glioma, prognosis

## Abstract

CD44 has been considered as a cancer stem cell marker in various tumors. With great enthusiasm, we read an article written by Wu et al. entitled “Expression of CD44 and the survival in glioma: a meta-analysis” published in *Bioscience Reports*. The authors performed meta-analyses to study the prognostic significance of CD44 in gliomas, and drew the conclusion that high expression of CD44 may predict poor survival in glioma, particularly in WHO grade II–III gliomas. However, two major defects exist in the present study, which made the meta-analysis on the prognostic significance of CD44 in all gliomas unreliable. In this commentary, we discussed the limitations and significance of the present study.

CD44 has been considered as a cancer stem cell marker in various tumors [1]. With great enthusiasm, we read an article written by Wu et al. entitled “Expression of CD44 and the survival in glioma: a meta-analysis” [2]. The authors performed the meta-analyses to study the prognostic significance of CD44 in gliomas and concluded that high expression of CD44 might predict poor survival in glioma, particularly in WHO grade II–III gliomas.

**Figure 1:**
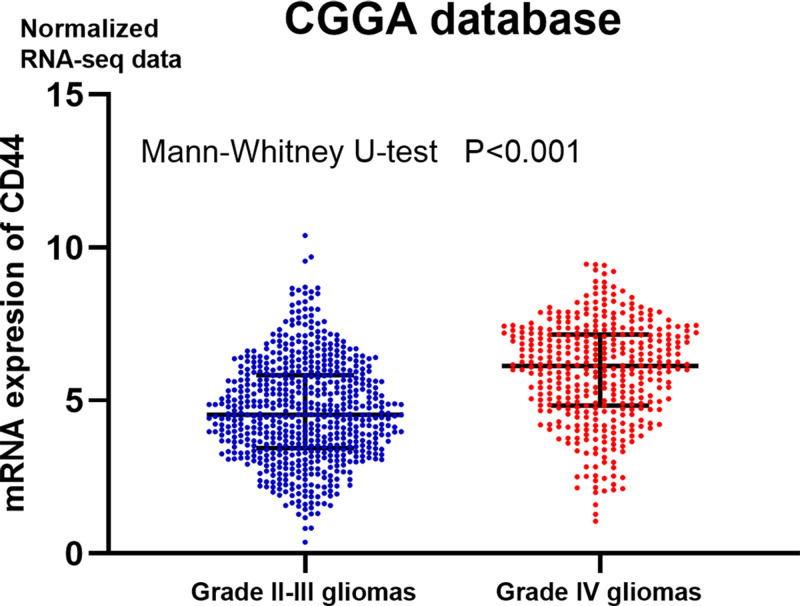
The mRNA expression levels of CD44 in the CGGA cohorts Abbreviation: CGGA, Chinese Glioma Genome Atlas.

Wu et al. [2] analyzed the prognostic significance of CD44 in gliomas. However, the major limitations of the present study are:

1. When analyzing the prognostic significance of CD44 in all gliomas, the hazard ratios (HRs) of CD44 protein expression and HRs of *CD44* mRNA expression were roughly combined. Different molecules (mRNA of *CD44*/protein of CD44) might represent different prognostic significance. Until now, no evidence has shown that the *CD44* mRNA and the CD44 protein expression have consistent tendencies in gliomas.
  Moreover, the cut-off values of the involved studies are not consistent. In 7/11 studies, the cut-off values were median, while in other studies, they were >70 or >75%. Different cut-off values represent different meanings of the HRs of CD44 expression in gliomas.
2. The HRs of CD44 in grade II–III gliomas was roughly combined with that of grade IV gliomas. First, the malignance of grade IV glioma is much higher than that of grade II–III gliomas. Second, the gene/protein expression profiles in grade II–III gliomas and grade IV gliomas differ significantly. For example, according to the normalized RNA-seq data in the Chinese Glioma Genome Atlas (CGGA), the mRNA expression of *CD44* in grade IV glioma is significantly higher than that in grade IV glioma (Figure 1). Thus, when examining the prognostic significance of CD44 in gliomas, the cut-off value of CD44 expression should be defined separately, depending on the grade of the gliomas. In summary, in this meta-analysis, HRs of CD44 in grade II–III gliomas should not be combined with that in grade IV gliomas.

The subgroup meta-analysis depending on the grade of the gliomas conducted by Wu et al. [2] is valuable. In analyzing the prognostic significance of CD44 mRNA expression in grade II–III gliomas, the two involved studies exhibit a high homogeneity [3,4]. We support the conclusion that high mRNA expression of *CD44* is associated with a poor prognosis of grade II–III gliomas. By combing the data from Hou et al. study [3] and the data from The Cancer Genome Atlas (TCGA) and CGGA databases, we performed a meta-analysis. The result (HR = 1.71, 95% confidence interval = 1.33–2.21) suggested that high expression of CD44 is associated with a poor prognosis in grade II–III gliomas. Interestingly, based on the TCGA and CGGA databases, we found that high mRNA expression of CD44 is not associated with poor overall survival of grade IV glioma (Figure 2). Besides, Klank et al. found a biphasic relationship between *CD44* expression levels and survival of high-grade glioma patients (the poorest outcomes occurring at intermediate levels) [5]. Thus, more studies are needed to further explore the relationship between CD44 gene/protein expression and grade IV gliomas.

**Figure 2:**
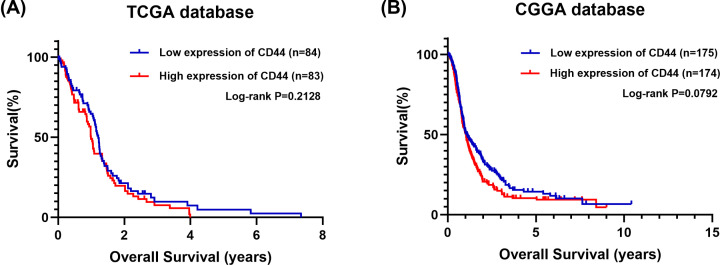
Correlation between CD44 mRNA expression and overall survival of grade IV glioma patients (**A**) The association between CD44 mRNA expression and OS in grade IV glioma patients in TCGA cohorts. (**B**) The association between CD44 mRNA expression and OS in grade IV glioma patients in CGGA cohorts. Abbreviation: OS, overall survival.

## Abbreviations

CGGA: Chinese Glioma Genome Atlas
HR: hazard ratio
TCGA: The Cancer Genome Atlas
